# Hypertension and undiagnosed hypertension among Bangladeshi adults: Identifying prevalence and associated factors using a nationwide survey

**DOI:** 10.3389/fpubh.2022.1066449

**Published:** 2022-12-06

**Authors:** Ahmed Hossain, Shakib Ahmed Suhel, Saifur Rahman Chowdhury, Shofiqul Islam, Nayma Akther, Nipa Rani Dhor, Mohammad Zakir Hossain, Mohammad Anwar Hossain, Syed Azizur Rahman

**Affiliations:** ^1^Health Services Administration, College of Health Sciences, University of Sharjah, Sharjah, United Arab Emirates; ^2^Department of Public Health, North South University, Dhaka, Bangladesh; ^3^Department of Health Research Methods, Evidence, and Impact (HEI), McMaster University, Hamilton, ON, Canada; ^4^Rangpur Hypertension and Research Center, Rangpur, Bangladesh

**Keywords:** hypertension, undiagnosed hypertension, non-communicable diseases, prevalence, Bangladesh, BDHS

## Abstract

**Background:**

Although undiagnosed hypertension (HTN) is a serious concern worldwide, it is less of an importance in Bangladesh, where there is a dearth of research on the subject. So, we aimed to identify the prevalence and associated factors for diagnosed and undiagnosed HTN.

**Methods:**

We analyzed the recent 2017–2018 Bangladesh Demographic and Health Survey data. We included 11,981 participants aged 18 years and above for the analysis. The prevalence rates of both diagnosed and undiagnosed hypertension were computed for all individuals and subgroups. The influence of socio-demographic, household, and community-related variables on HTN and undiagnosed HTN was investigated using multinomial regression analysis.

**Results:**

The study finds 1,464 (12.2%) of the 11,981 respondents [6,815 females [56.9 %]; mean age 39.4 years] had diagnosed HTN, whereas 1 898 (15.8%) had undiagnosed HTN. The HTN and undiagnosed HTN were significantly prevalent in the elderly, type 2 diabetic (T2DM), and overweight and obese individuals. In terms of residential regions, people from coastal region had a significantly higher prevalence of both HTN (RRR: 1.37; 95% CI: 1.17–1.62) and undiagnosed HTN (RRR: 1.35; 95% CI: 1.17–1.56) compared to those from the central region of Bangladesh.

**Conclusions:**

The high prevalence of undetected hypertension in Bangladesh suggests that screening procedures for the current chronic illness may be inadequate in routine clinical practice. All populations should have access to hypertension screening, but it is especially crucial for the elderly, those with diabetes, those who are overweight or obese, and those from coastal and northern regions of Bangladesh.

## Introduction

Hypertension, or consistently high blood pressure, is a serious medical condition that raises the risk of many different diseases, including those affecting the heart, brain, and kidneys (1). The World Health Organization estimates that 1.28 billion adults aged 30–79 years worldwide have hypertension, most (two-thirds) living in low- and middle-income countries (1). Moreover, non-communicable diseases, including hypertension, claimed the lives of 41 million individuals worldwide, accounting for 74% of all deaths (2). Undiagnosed hypertension is another health concern because many individuals are not receiving medication to lower their high blood pressure because they are unaware that it exists. According to a survey, more than half of hypertensive patients are ignorant of their hypertensive condition (3). The high global burden of untreated hypertension emphasizes the necessity of early identification and improved hypertension screening. The prevention and control of hypertension can significantly contribute to reaching Sustainable Development Goal 3.4 on non-communicable diseases (4).

Hypertensive heart disease is a long-term condition that worsens with time. Several risk factors, including advancing age, family history, obesity, high salt diets (more than 3 g per day), physical inactivity, and excessive alcohol intake, have strong and independent connections with the onset of hypertension (5–7). In Bangladesh, roughly 68% of deaths are caused by non-communicable diseases, with hypertension accounting for 15–20 % (8). Hypertension in Bangladesh is the leading modifiable cause of a variety of health problems and a resultant economic burden (9, 10).

Heart disease caused by hypertension can result in diastolic failure, systolic failure, or a mix of the two (5). Patients with untreated hypertension are more likely to have acute consequences such as decompensated heart failure, acute coronary syndrome, and sudden cardiac death (11, 12). Several studies have approximated the prevalence of hypertension in Bangladesh; however, the prevalence of undiagnosed hypertension remains uncertain (13). According to a study conducted in 2005, the prevalence of undiagnosed hypertension was 11.1%, rising with age to 22.7% among those aged 60 and older (14).

Those who are aware of their hypertension often seek treatment, whereas those whose hypertension is undiagnosed may go untreated for years. Since patients with diagnosed hypertension have the option of hypertension medication to reduce their risk of adverse outcomes, a delay in diagnosis can be fatal. Cardiovascular disease, coronary artery disease, stroke, renal failure, and disability have been identified in more than 50% of newly diagnosed hypertensive patients (15, 16). In order to limit the number of deaths, healthcare visits, and costs related to hypertension, it is vital to diagnose the problem early (17). This analysis of recent national survey data for prevalence estimation and identifying associated factors advises that policymakers address the issue of undiagnosed hypertension in order to reduce the burden of hypertension-related health outcomes.

## Materials and methods

### Study design and setting

**Figure 1:**
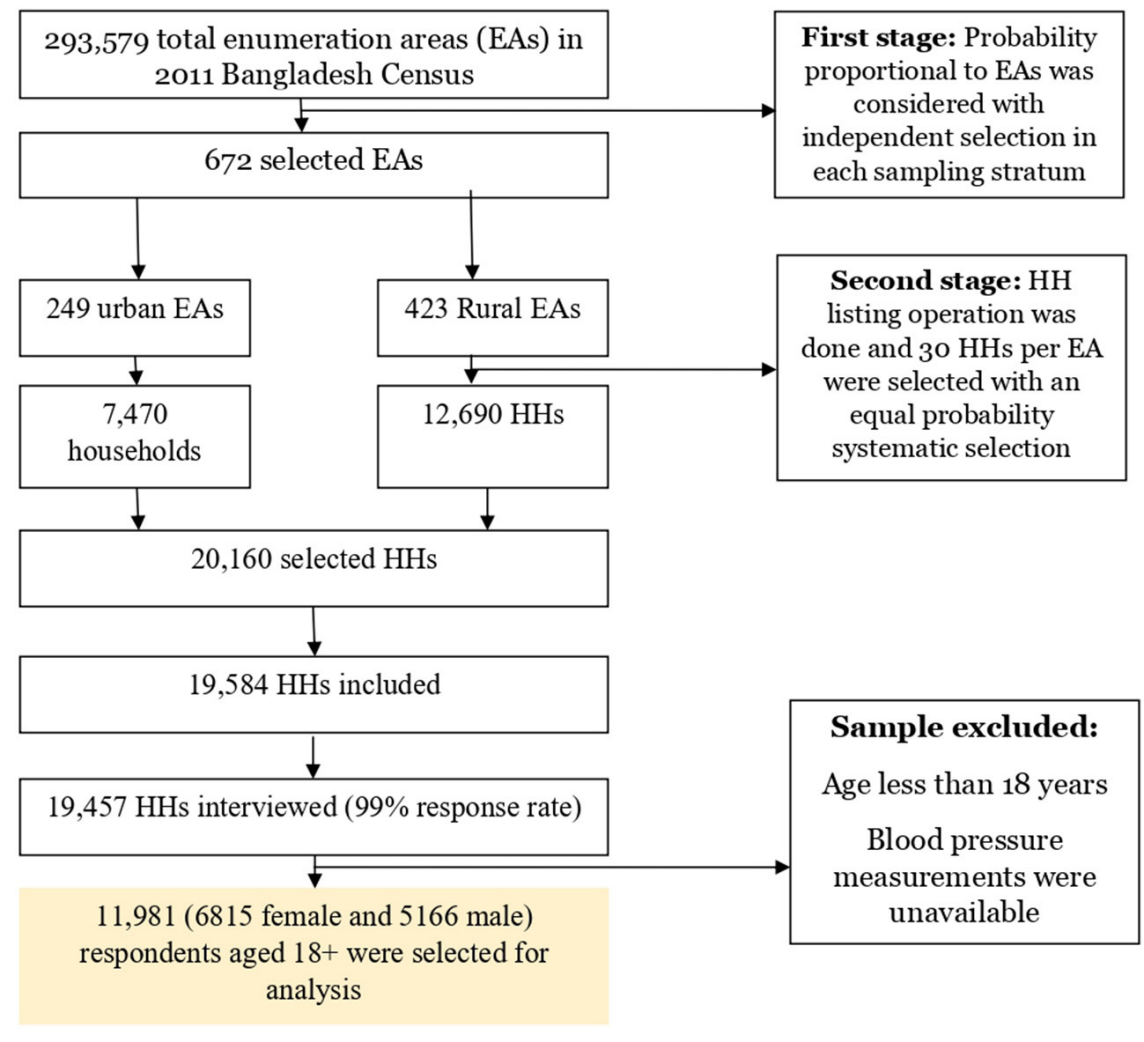
Flowchart of selecting study participants.

We analyzed the data from the Bangladesh Demographic and Health Survey (BDHS) 2017–2018, which applied a cross-sectional survey from October 2017 to March 2018. The sample for the BDHS is nationally representative and covers the entire population residing in non-institutional dwelling units in the country. The survey used a list of enumeration areas (EAs) from the 2011 Bangladesh census as a sampling frame. The primary sampling unit (PSU) of the survey is an EA with an average of about 120 households. Figure 1 provides a flowchart detailing the process of selecting study participants. The BDHS used a stratified sample of households through two stages, with strata for rural and urban regions. In the first stage, 675 PSUs were chosen, with probability proportional to PSU size. The study contained 672 PSUs (three PSUs were not sampled due to floods), with 249 PSUs from urban regions and 423 PSUs from rural areas. A complete household listing operation was then carried out in all selected PSUs to provide a sampling frame for the second-stage selection of households. The second stage involved randomly selecting (systematic) about 30 households in each PSU to provide statistically valid estimates of health outcomes for the country, each of the eight divisions, and urban and rural areas individually. A total of 19 457 of the 19 584 eligible households were interviewed, resulting in a household response rate of 99.4%. Blood pressure (BP) measures were collected from all women and men aged 18 and above in the subsample of one-fourth of the households. Thus, we included 11 981 adult participants in the analysis after removing the missing information on blood pressure and participants < 18 years. More details about survey sampling and data collection techniques can be found in the BDHS survey report (18).

### Outcome measure

According to the Seventh Report of the Joint National Committee (JNC) on Prevention, Detection, Evaluation, and Treatment of High Blood Pressure, a person with SBP ≥140 mmHg and/or DBP ≥90 mmHg and/or currently on treatment with antihypertensive medication was identified as having hypertension (11). Undiagnosed hypertension was defined as not known to have hypertension but having an average SBP ≥140 mmHg and/or a DBP ≥90 mmHg and never taking prescribed medicine or being counseled by health experts to decrease or regulate blood pressure (7, 14). According to BDHS guidelines, the enumerators assessed blood pressure three times 10 min apart, and the definition of hypertension was determined by averaging the last two measurements. The LIFE SOURCE^®^ UA-767 Plus BP monitor was used to measure blood pressure. This automatic device includes separate cuffs (small, medium, and large). Two health technicians were trained per team according to the recommended protocol to measure blood pressure.

### Independent variables

Explanatory variables were included at the individual, household, and community levels. Participants' age in years (< 30, 30–39, 40–49, 50–59, 60–69, ≥70), sex (Male, Female), marital status (Never married, Married, Widowed/Divorced/Separated), education (No education, Primary, Secondary, and Higher-secondary and above), body mass index (BMI) (Underweight: BMI < 18.5 kg/m^2^, Normal: BMI 18.5–24.99 kg/m^2^, Overweight: BMI 25–29.99 kg/m^2^, and Obese: BMI ≥30 kg/m^2^). According to the WHO classification, people with a fasting blood glucose ≥ 7.0 mmol/L (126 mg/dl) in the presence of signs and symptoms are considered to have diabetes (19). We defined diabetes following WHO classification and taking into consideration of medication use for T2DM. The wealth status was a household-level element classified as Poor, Middle, and Rich group. It was produced using principal component analysis (PCA) from a household's different assets (e.g., televisions, bicycles, drinking water sources, sanitation facilities, and building materials) (20). The community-level factors included the place of residence (City, Semi-urban, and Rural). The residential region of the country is classified based on its administrative divisions. We considered Barisal, Chattogram, and Khulna to be in the coastal region, Dhaka and Mymensingh in the central region, Rajshahi and Rangpur in the North region, and Sylhet in the East region.

### Data analysis

For the analysis, we utilized the statistical program R 4.1.3. The BDHS provided a survey weight for each individual record, which was used to adjust the sample size of the resulting statistics so that they were more broadly representative of the population. Categorical data were given as numbers and percentages, and continuous variables were provided as mean and standard deviation. Baseline characteristics were summarized according to normal, diagnosed, and undiagnosed hypertension, as well as by subgroups. To investigate the associated variables, a multivariable multinomial regression model was used to adjusting for covariates. Because of the importance that each variable has as a covariate, we decided to include them all in the regression model. All the variables in the multinomial logistic regression model were entered in one step. The outcome variable had three categories representing participants with normal, diagnosed, and undiagnosed hypertension. The normal category was considered as the reference category in the regression analysis to which the other two categories were compared. The model's multinomial regression coefficient was exponentiated and reported as relative risk ratios (RRR) with 95% confidence intervals (CI). Relative risk ratio (RRR) is defined as the ratio of the probability of an outcome in the exposed group to the probability of an outcome in the unexposed group (21). A post-estimation test, variance inflation factor (VIF), was performed to determine the multi-collinearity. In this current study, a *p*-value < 0.05 was considered statistically significant.

## Results

### Characteristics of the study participants

In the analysis, 11,981 respondents were included, and their socio-demographic characteristics are shown in Table 1. The mean age of the participants was 39.43 (SD: 16.04) years. About 33% of respondents were under 30 years of age, 15% were 60 years or older, and 56.9% were female. Among the participants, 25% lacked any formal education, 30.4% had completed primary school, and the remaining 44.6% had completed secondary school or greater. The majority of participants, 7,697 (64.2%), were from rural areas, while 3,202 (26.7%) were from semi-urban areas. The 2,910 participants (24.3%) were either overweight or obese. It seems that 1,009, (8.4%) of respondents were diabetic. The Table 1 shows 4,542 (37.9%) of the participants were from Bangladesh's coastal region, while 2,861 (23.9%) were from the central region, and 3,155 (26.3%) were from the northern region of Bangladesh.

**Table 1:** Distribution of normal, hypertensive, and undiagnosed hypertensive people by socio-demographic groups of the respondents.

**Variables**	**Normal (*n =* 8,619, 72%)**	**Diagnosed hypertensive (*n =* 1,464, 12.2%)**	**Undiagnosed hypertensive (*n =* 1,898, 15.8%)**	**Total = 11,981**
**Sex**
Female	4,842 (71%)	992 (14.6%)	981 (14.4%)	6,815 (56.9%)
Male	3,777 (73.1%)	472 (9.1%)	917 (17.8%)	5,166 (43.1%)
**Age in years**
< 30	3,509 (89.6%)	100 (2.6%)	306 (7.8%)	3,915 (32.7%)
30–39	2,184 (76.5%)	227 (8%)	443 (15.5%)	2,854 (23.9%)
40–49	1,337 (65.3%)	325 (15.9%)	384 (18.8%)	2,046 (17.1%)
50–59	754 (56%)	304 (22.6%)	289 (21.5%)	1,347 (11.3%)
60–69	528 (48.7%)	300 (27.6%)	257 (23.7%)	1,085 (9.1%)
≥70	300 (42.2%)	202 (28.4%)	209 (29.4%)	711 (5.9%)
**Marital status**
Never married	1,097 (88%)	13 (1%)	136 (10.9%)	1,246 (10.4%)
Married	6,952 (72.6%)	1,149 (12%)	1,481 (15.5%)	9,582 (80%)
Widowed/divorced	570 (49.4%)	302 (26.2%)	281 (24.4%)	1,153 (9.6%)
**Education**
No education	1,932 (64.3%)	481 (16%)	589 (19.6%)	3,001 (25%)
Primary	2,625 (72.2%)	451 (12.4%)	562 (15.4%)	3,638 (30.4%)
Secondary	2,644 (76.3%)	353 (10.2%)	467 (13.5%)	3,464 (28.9%)
Higher-secondary and above	1,419 (75.6%)	179(9.5%)	280 (14.9%)	1,878 (15.7%)
**Currently working**
No	3,173 (68.2%)	746 (16%)	731 (15.7%)	4,650 (38.8%)
Yes	5,446 (74.3%)	718 (9.8%)	1,167(15.9%)	7,331 (61.2%)
**Wealth status**
Poor	1,705 (74.8%)	217 (9.5%)	356 (15.6%)	2,278 (19%)
Middle	1,726 (72.8%)	276 (11.6%)	370 (15.6%)	2,372 (19.8%)
Rich	5,188 (70.8%)	971 (13.2%)	1,172 (16%)	7,331 (61.2%)
**Diabetic**
No	8,071 (73.6%)	1,210 (11%)	1,691 (15.4%)	10,972 (91.6%)
Yes	548 (54.3%)	254 (25.2%)	207 (20.5%)	1,009 (8.4%)
**BMI**
Normal	5,278 (75.2%)	716 (10.2%)	1,021 (14.6%)	7,015 (58.6%)
Underweight	1,677 (81.6%)	122 (5.9%)	257 (12.5%)	2,056 (17.2%)
Overweight	1,394 (57.8%)	501 (20.8%)	516 (21.4%)	2,411 (20.1%)
Obese	270 (54.1%)	125 (25.1%)	104 (20.8%)	499 (4.2%)
**Residence**
City	769 (71.1%)	152 (14%)	161 (14.9%)	1,082 (9%)
Semi-urban	2,253 (70.4%)	447 (14%)	502 (15.7%)	3,202 (26.7%)
Rural	5,597 (72.7%)	865 (11.2%)	1,235 (16%)	7,697 (64.2%)
**Residential region**
Central	2,185 (76.4%)	300 (10.5%)	376 (13.1%)	2,861 (23.9%)
Coastal	3,149 (69.3%)	636 (14%)	757 (16.7%)	4,542 (37.9%)
North	2,231 (70.7%)	339 (10.7%)	585 (18.5%)	3,155 (26.3%)
East	1,054 (74.1%)	189 (13.3%)	180 (12.6%)	1,423 (11.9%)

### Prevalence of diagnosed HTN and undiagnosed HTN

Table 1 shows the prevalence of both diagnosed and undiagnosed hypertension in study participants. Among the participants, 1,464 (12.2%) of participants were hypertensive, while 1,898 (15.8%) had undiagnosed hypertension.

Table 1 reveals that diagnosed HTN was higher among females (14.6 vs. 9.1%), while undiagnosed HTN (14.4 vs. 17.8%) was found to be lower among females compared to their male counterparts. With the increase of age, the prevalence of both HTN and undiagnosed HTN increased, while the highest prevalence was found among the age group of ≥70 years. The prevalence of both diagnosed and undiagnosed hypertension was high among those with no formal education. Individuals from wealthy homes showed greater rates of both diagnosed (13.2%) and undiagnosed hypertension (16.0%). Importantly, persons with diabetes had a high prevalence of both recognized (25.2%) and undiagnosed hypertension (20.5%). Those with a normal BMI were shown to have a lower prevalence of both diagnosed and undiagnosed hypertension compared to those who were overweight or obese. Among the various residential regions of Bangladesh, the prevalence of diagnosed HTN was highest in the coastal region (14.0%), whereas the prevalence of undiagnosed HTN was highest in the northern region (18.5%).

**Figure 2:**
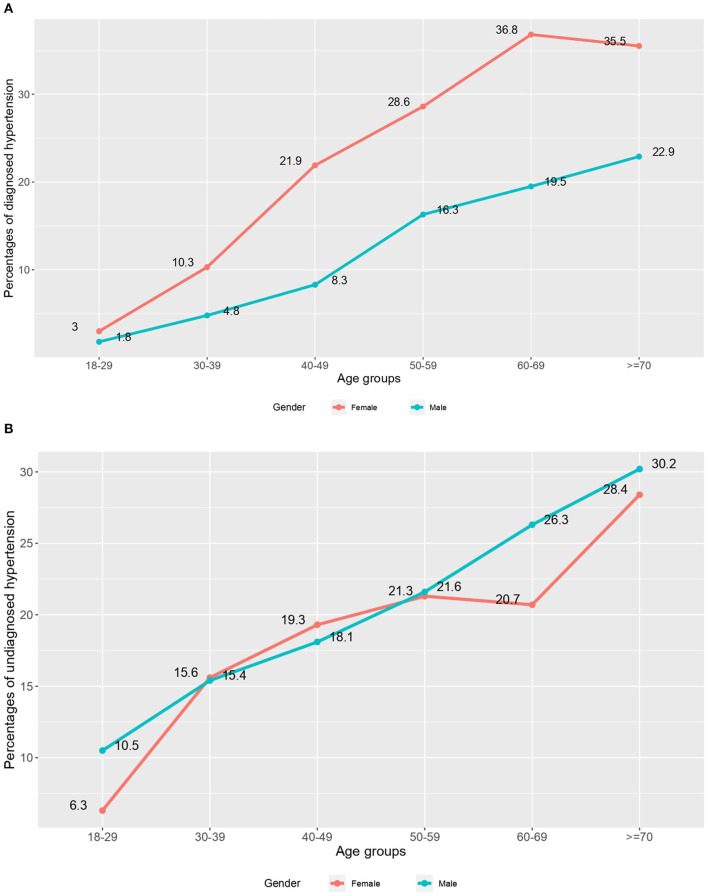
**(A)** Prevalence of diagnosed hypertension by age and gender. **(B)** Prevalence of undiagnosed hypertension by age and gender.

Figures 2A,B show the prevalence of diagnosed and undiagnosed hypertension by age and gender, respectively. It shows that females were diagnosed more with hypertension than male individuals at any age. After the age of 50, undiagnosed hypertension became widespread in males, as shown in Figure 2B. Undiagnosed hypertension became more common with age, and it was especially common among older men.

### Results of multinomial regression analysis

The results from adjusted multinomial logistic regression models for predicting both diagnosed and undiagnosed hypertension is presented in Table 2. Males were 55% less likely than females to be diagnosed with hypertension [Relative Risk Ratio (RRR) = 0.45, 95% Confidence Interval (CI) = 0.39–0.51]. People in older age groups had significantly higher rates of both diagnosed and undiagnosed hypertension than those between the ages of 30 and 39. For instance, the likelihood of having diagnosed HTN (RRR = 14.51, 95% CI = 11.24–18.71) and undiagnosed HTN (RRR = 4.66, 95% CI = 3.74–5.81) was 14.51 and 4.66 times greater among people aged ≥70 years compared to those aged 30–39 years, respectively. Education has a vital role in having been diagnosed with hypertension. For instance, those with secondary education or higher had a 50% higher risk of HTN diagnosis than those with no formal education (RRR = 1.50, 95% CI = 1.19–1.90). Diabetes was associated with a higher risk of both diagnosed (RRR = 1.57, 95% CI = 1.23–2.0) and undiagnosed hypertension (RRR = 1.35, 95% CI = 1.13–1.62) compared to patients without diabetes. Compared to individuals with a normal body mass index, participants categorized as overweight or obese had a greater likelihood of having both diagnosed and undiagnosed hypertension. Obese persons had 3.07 times (RRR = 3.07, 95% CI = 2.37–3.98) and 2.16 times (RRR = 2.16, 95% CI = 1.68–2.77) greater risk of diagnosed HTN and undiagnosed HTN, respectively, compared to normal-weight individuals. Participants from the coastal region (RRR = 1.37, 95% CI = 1.17–1.62) and the northern region (RRR = 1.64, 95% CI = 1.32–2.04) were 37% and 64% more likely to have been diagnosed with HTN, respectively than those from the central region of Bangladesh. Northern (RRR = 1.56, 95% CI = 1.35–1.81), and coastal (RRR = 1.35, 95% CI = 1.17–1.56) participants had a 56% and 35% higher risk of undiagnosed HTN, respectively than those from the central region.

**Table 2:** Associated factors of diagnosed hypertension and undiagnosed hypertension identified using multivariable multinomial regression models.

	**Diagnosed hypertension vs. normal**		**Undiagnosed hypertension vs. normal**	
	**RRR**	**95%CI**	**RRR**	**95%CI**
**Sex (ref: Female)**
Male	0.45	**0.39–0.51**	1.06	0.95–1.18
**Age group (ref: 30–39)**
< 30	0.30	**0.24–0.39**	0.49	**0.41–0.57**
40–49	2.77	**2.28–3.36**	1.48	**1.12–1.73**
50–59	5.89	**4.78–7.26**	2.18	**1.82–2.60**
60–69	10.09	**8.08–12.58**	2.98	**2.46–3.61**
≥70	14.51	**11.24–18.71**	4.66	**3.74–5.81**
**Education (ref: No education)**				
Primary	1.22	**1.03–1.43**	0.99	0.86**–**1.14
Secondary	1.36	**1.13–1.64**	0.98	0.84**–**1.15
Higher secondary and above	1.50	**1.19–1.90**	1.11	0.92**–**1.35
**Wealth status (ref: Poor)**				
Middle	1.10	0.90**–**1.36	0.99	0.83**–**1.17
Rich	1.12	0.94**–**1.34	1.02	0.88**–**1.17
**Diabetic (ref: No)**
Yes	1.82	**1.52–2.19**	1.35	**1.13–1.62**
**BMI (ref: Normal)**
Underweight	0.40	**0.32–0.50**	0.65	**0.55–0.76**
Overweight	2.73	**2.36–3.17**	2.09	**1.83–2.38**
Obese	3.07	**2.37–3.98**	2.16	**1.68–2.77**
**Residence (ref: City)**
Semi-urban	1.29	1.02–1.63	1.12	0.91–1.38
Rural	0.98	0.79–1.23	1.10	0.90–1.33
**Residential region (ref: Central)**
Coastal	1.37	**1.17**–**1.62**	1.35	**1.17**–**1.56**
North	1.19	0.99–1.43	1.56	**1.35**–**1.81**
East	1.64	**1.32**–**2.04**	1.11	0.91–1.36

95% Confidence Intervals (CIs) appearing in bold are statistically significant with a *p*-value < 0.05.

## Discussion

This study evaluates the prevalence of diagnosed hypertension, undiagnosed hypertension, and their contributing variables among Bangladeshi adults. According to the survey, 28% of Bangladeshis had hypertension, and we found 16% had undiagnosed hypertension and 12% had diagnosed hypertension. The overall hypertension prevalence is similar to the prevalence in other countries like China (26.6%) (22), Italy (25.9%) (23), Australia (25.9%) (24). Study from India (40.6%) (25) showed a high prevalence of hypertension, and Mexico (17.2%) (26) showed a low prevalence.

Moreover, the rate of undetected hypertension in our study was comparable to those research conducted in Ethiopia (12.7%) (27), and Ghana (18.5%) (28). Other studies found a higher prevalence of undiagnosed hypertension in Western India (26%) (29), Ethiopia (24.8%) (30), and Finland (24%) (31). A recent study conducted in Bangladesh found that among hypertensive patients, 36.7% were aware that they had the condition, and only 31.1% received anti-hypertensive medications (32). This finding demonstrates a low treatment-seeking behavior among hypertensive people in the country.

Hypertension is common in elderly people, and undiagnosed hypertension increases with age. In line with earlier research, our data showed that the risk of undetected hypertension was high among older person (33–35). Overall prevalence of undiagnosed hypertension among men and women was similar. But men over the age of 50 were more likely than women of the same age to have undiagnosed hypertension, which may indicate that older men had lower levels of awareness, and/or participation in early detection initiatives. This is in line with the findings of a study that found older males were less likely to be aware of their hypertension diagnosis, receive treatment for it, or have it under control than women of the same age (35).

Similar to earlier research on hypertension and undiagnosed hypertension, studies from Bangladesh, and Nepal demonstrate a substantial correlation between wealth index and hypertension (36–38). The wealthiest individuals revealed a high prevalence of both hypertension and undetected hypertension in this study. People from the wealthiest families often consumed more calories, which was exacerbated by their propensity to lead sedentary lifestyles and develop hypertension (39).

The prevalence of hypertension is significantly higher among residents of Bangladesh's coastal and eastern regions in our study, and these factors are significantly linked to both hypertension and undiagnosed hypertension, which is consistent with results from a previous study in Bangladesh (40).

Our findings have important policy implications, suggesting that early detection and screening are essential for reducing the prevalence of undiagnosed hypertension in Bangladesh. Second, the prevention of undetected hypertension in Bangladesh depends critically on effective weight control and the reduction of obesity. With a strong correlation between age and undetected hypertension, rising life expectancy in Bangladesh (now 72.3 years) may lead to an even greater future burden of undiagnosed hypertension (41). A possible explanation for the high prevalence of undetected hypertension is that screening procedure for the current condition is ineffective in routine clinical practice. Healthcare resources should be allocated such that people from all wealth quintiles have similar access to them in order to lessen the gap in the rate of undetected hypertension. More robust health promotion measures are required to improve the prevalence of undiagnosed hypertension, especially among men in the coastal and northern regions of Bangladesh.

The study's strength is its use of a large, nationally representative data, which increases the possibility of its external validity. In addition, clinical factors such as glucose at fasting, blood pressure, body weight, and height were objectively examined. Our multinomial logistic regression approach rectified the problem of traditional logistic regression's overestimation of effect size and boosted the precision of our findings. The inability to draw firm conclusions about cause and effect from this cross-sectional study is a major limitation. Due to the unavailability of data on potential confounders for developing hypertension, such as the family history of hypertension, lifestyle, dietary practice, physical activity status, and medication history, these variables could not be incorporated into the study. Furthermore, the data of a significant number of variables were self-reported, so the survey had to rely on participants' responses, which could introduce the possibility of response bias.

## Conclusions

A high prevalence of undiagnosed hypertension is found in Bangladesh. The prevalence of undiagnosed hypertension increased with age, and a significantly higher prevalence was observed in the older male groups. People who are elderly, those who have diabetes, those who are overweight or obese, and those who live in coastal regions are all contributing variables that might lead to hypertension going untreated. The Sustainable Development Goals (SDG) include a specific goal of a 25% relative reduction in the prevalence of raised blood pressure by 2030. Reducing the proportion of undiagnosed hypertension in a country is critical to achieving this goal. Screening measures for the current illness may be inadequate in routine clinical practice, as evidenced by the high prevalence of undiagnosed hypertension in Bangladesh. Screening for hypertension should be equally available to all populations, but it is especially important for the elderly, persons with diabetes, and those who are very overweight or obese. Policymakers and government officials should prioritize coastal and northern adult populations in their efforts to minimize the prevalence of undiagnosed hypertension.

## Data availability statement

The original contributions presented in the study are included in the article/supplementary material, further inquiries can be directed to the corresponding author/s.

## Ethics statement

Ethical review and approval was not required for the study on human participants in accordance with the local legislation and institutional requirements. The patients/participants provided their written informed consent to participate in this study.

## Author contributions

AH, SS, SI, and SC contributed to the literature search and study concept and design. AH, SS, SI, SC, NA, and ND contributed to the data acquisition. AH and SC accessed the data and contributed to the data analysis. AH, SS, MZH, MAH, and SR contributed to the data interpretation. AH, SS, SC, MZH, and SR drafted the manuscript. All authors contributed to the critical revision of the manuscript.

## Conflict of interest

The authors declare that the research was conducted in the absence of any commercial or financial relationships that could be construed as a potential conflict of interest.

## Publisher's note

All claims expressed in this article are solely those of the authors and do not necessarily represent those of their affiliated organizations, or those of the publisher, the editors and the reviewers. Any product that may be evaluated in this article, or claim that may be made by its manufacturer, is not guaranteed or endorsed by the publisher.
